# Complete Neurodevelopmental and Cardiac Recovery After Prolonged Cardiac Arrest With Extracorporeal Resuscitation: A Case Report

**DOI:** 10.7759/cureus.98914

**Published:** 2025-12-10

**Authors:** Prashant J Purohit, Len Y Tanaka, Sheree Kuo, Allison A Ohigashi, Melissa Yamauchi, András Bratincsák

**Affiliations:** 1 Pediatrics/Pediatric Critical Care, Kapiolani Medical Center for Women and Children, Honolulu, USA; 2 Pediatrics, University of Hawaii John A. Burns School of Medicine, Honolulu, USA; 3 Pediatric Critical Care, Kapiolani Medical Center for Women and Children, Honolulu, USA; 4 Neonatology, Kapiolani Medical Center for Women and Children, John A. Burns School of Medicine, Honolulu, USA; 5 Pharmacology, Kapiolani Medical Center for Women and Children, Honolulu, USA; 6 Pediatric Cardiology, Kapiolani Medical Center for Women and Children, Honolulu, USA

**Keywords:** cardiac arrest, cpr, ecls, ecmo, extracorporeal resuscitation, limb ischemia, neurodevelopmental outcome

## Abstract

Acute fulminant myocarditis (AFM) is characterized by severe myocardial inflammation associated with cardiogenic shock, cardiac arrest, and a high mortality risk. Early extracorporeal life support (ECLS) can improve survival but also carries the risk of serious complications. We present the case of a two-year-old female patient admitted with AFM and cardiogenic shock. While preparations were underway to initiate ECLS, she suffered a sudden cardiac arrest. The patient underwent extracorporeal cardiopulmonary resuscitation (ECPR) for 27 minutes. She remained in a state of myocardial stunning for the next two days. Her clinical course was further complicated by the development of a large intracardiac thrombus, which required systemic thrombolysis with alteplase and anticoagulation therapy. She progressed to multiorgan failure, fluid overload, and rhabdomyolysis, necessitating continuous renal replacement therapy (CRRT). The patient remained on ECLS for 20 days and CRRT for seven weeks. Despite a prolonged and complex course, she was ultimately discharged home with an excellent neurodevelopmental outcome and a complete cardiac recovery. This case highlights key factors that may contribute to improved outcomes in pediatric patients with AFM requiring ECPR and ECLS.

## Introduction

Extracorporeal life support (ECLS) provides temporary cardiopulmonary bypass for critically ill patients with reversible conditions that are unresponsive to conventional measures, including mechanical ventilation, fluid resuscitation, and inotropic support [1]. Extracorporeal cardiopulmonary resuscitation (ECPR) is defined as the initiation of ECLS during conventional cardiopulmonary resuscitation (CCPR) or within 20 minutes of return of spontaneous circulation (ROSC) [2,3]. Mortality rates are very high when CCPR extends beyond 30-35 minutes. Previous literature reports a mortality rate of 88%-100% [4,5]. In contrast, ECPR has achieved survival even when resuscitation efforts exceeded 90 minutes [4]. These observations highlight the potential advantage of ECPR over CCPR in prolonged resuscitation, although the optimal duration and clear endpoints remain undefined. Recognizing that favorable outcomes are possible even after extended ECPR is important in guiding clinical decision-making.

ECLS outcomes are influenced by patient demographics, underlying pathology, illness severity, extracorporeal membrane oxygenation (ECMO) mode, timing of initiation, duration of support, and associated comorbidities [1-9]. Patients experiencing cardiac arrest demonstrate significant clinical complexity and are susceptible to complications, including myocardial stunning and intracardiac thrombus formation.

Myocardial stunning is characterized by severe myocardial contractile dysfunction following acute ischemia and has been associated with poor prognosis, including survival as low as 0.6% in one report [7].

We report the case of a two-year-old female patient with acute fulminant myocarditis (AFM) who developed cardiac arrest requiring 27 minutes of ECPR. Despite myocardial stunning, intraventricular thrombus formation, and acute renal failure, she survived to discharge with complete cardiac and renal recovery and excellent neurodevelopmental outcome.

A part of this work was previously presented as a poster titled “Use of systemic tissue plasminogen activator for intraventricular thrombus on VA ECMO” at the 36th Annual Children’s National Medical Center Symposium on ECMO and Advanced Therapies for Respiratory Failure, Keystone, CO, 2020.

## Case presentation

A two-year-old female patient with a known secundum atrial septal defect (ASD) and a solitary right kidney presented after three days of rhinorrhea, one day of fatigue, and acute respiratory distress on the morning of admission. She had been in her usual state of health prior to this illness.

In the emergency department (ED), her temperature was 100.4°F, heart rate was 158/minute, respiratory rate was 28/minute, blood pressure was 101/61 mmHg, oxygen saturation was 94% on room air, and capillary refill time was 2-3 seconds. Examination revealed bilateral periorbital edema, tachypnea with retractions, hepatomegaly, and a grade 1-2/6 systolic murmur at the left upper sternal border. She received two 10 mL/kg slow fluid boluses and was started on high-flow nasal cannula (15 L/minute, FiO₂ 40%).

Initial laboratory tests, as summarized in Table 1, were significant for mildly elevated blood urea nitrogen (BUN), anion gap, and N-terminal pro-B-type natriuretic peptide (NT-proBNP). The rest of the tests, including hepatic function test, streptozyme, parvovirus, and respiratory virus polymerase chain reaction (PCR), were unremarkable. An electrocardiogram (ECG) showed sinus tachycardia, right axis deviation, low voltage in the left precordial leads, and minimal ST elevation in lead I. An echocardiogram revealed a large pericardial effusion with intermittent right atrial collapse, left ventricular ejection fraction (LVEF) of 66.4%, thickened myocardium, moderately dilated and mildly hypertrophied right ventricle (RV), mildly reduced RV systolic function, and an 8.8 mm secundum ASD.

**Table 1:** Diagnostic workup upon admission WBC: white blood cell, CRP: C-reactive protein, BUN: blood urea nitrogen, Na: sodium, K: potassium, CO₂: carbon dioxide, CK: creatine kinase, NT-proBNP: N-terminal pro-brain natriuretic peptide, ASO: antistreptolysin O

Test	Result	Reference value
WBC count	4 × 10⁹/L	6-17 × 10⁹/L
Hemoglobin	15.2 g/dL	11-14 g/dL
Platelet count	191 × 10⁹/L	204-402 × 10⁹/L
CRP	1.8 mg/L	<5 mg/dL
Procalcitonin	0.41 ng/mL	<0.10 ng/mL
BUN	37 mg/dL	8-24 mg/dL
Creatinine	0.55 mg/dL	0.4-1.1 mg/dL
Na	131 mmol/L	136-145 mmol/L
K	4.5 mmol/L	3.3-5.1 mmol/L
Chloride	91 mmol/L	96-108 mmol/L
CO₂	14 mmol/L	21-31 mmol/L
Glucose	113 mg/dL	70-99 mg/dL
Calcium	9.1 mg/dL	8.8-10.8 mg/dL
Anion gap	26	9-18
CK	204 U/L	48-240 U/L
NT-proBNP	2,125 pg/mL	0-125 pg/mL
ASO	<20 IU/mL	<100 IU/mL

She was admitted to the pediatric intensive care unit (PICU) and underwent urgent pericardiocentesis. The pericardial fluid analysis showed a slightly hazy yellow appearance, with 48 WBCs (1 band, 1 neutrophil, 32 lymphocytes, and 66 monocytes), 101 RBCs, glucose at 90 mg/dL, and lactate dehydrogenase (LDH) at 226 U/L. Blood testing was negative for adenovirus, cytomegalovirus, Epstein-Barr virus, enterovirus, and parvovirus.

The patient’s hemodynamics continued to decline despite pericardiocentesis, necessitating support with epinephrine, milrinone, and calcium infusions. Based on the worsening clinical condition and worsening LVEF from 66.4% to 48% within eight hours of admission on the repeat echocardiogram, she was started on methylprednisolone and intravenous immunoglobulin (IVIG) for presumed myocarditis. Sedation was maintained with dexmedetomidine infusion. Her further course, including workup and management, is summarized in Table 2 in chronological order. The values represent workup done at the indicated time points (±1 hour).

**Table 2:** Flow table of diagnostics and interventions HCO3: bicarbonate, ECHO: echocardiogram, LVEF: left ventricular ejection fraction, ECLS: extracorporeal life support, V-A ECLS: venoarterial extracorporeal life support, LV: left ventricle, LVSF: left ventricular shortening fraction

Time	pH	HCO3 (mmol/L)	Base deficit (mmol/L)	Lactate (mmol/L)	Troponin (ng/L)	ECHO	Intervention
0 hour	7.44 (v)	17.2	-5.2	3.53	43	Pericardial effusion, LVEF: 66.40%	Pericardiocentesis
10 hours	7.31	18.4	-7.1	5.26	Not done	LVEF: 48% (8 hours)	Intubation, arterial line placement
12 hours	7.49	18.6	-3.0	4.27	374	Not done	Continued care
14 hours	7.51	21.1	-0.2	4.40	Not done	Not done	Continued care
16 hours	7.27	17.4	-8.8	6.24	524	Not done	ECLS activation
18 hours	7.26	15.5	-10.5	6.68	Not done	Not done	Pre-arrest
19 hours	7.08	12.5	-16.7	13.12	Not done	Not done	Post-arrest, on V-A ECLS
Day 1	7.28	19.9	-6.7	8.17	33,521	Myocardial stun, sluggish flow versus LV thrombus	Continued care
Day 2	7.36	19.3	-5.2	1.38	23,784	Myocardial stun, LV thrombus	Initiation of bivalirudin and alteplase infusion
Day 3	7.43	23.7	0	2.47	Not done	LVSF: 4% (not LVEF), resolution of LV thrombus	Continued bivalirudin, completed alteplase; transferred to a higher center

Within six hours of admission, her lactate increased along with clinical deterioration. Therefore, she was intubated and had femoral arterial and venous lines placed. An institutional “ECLS heads-up” was activated for high-risk myocarditis. Sedation was escalated with morphine and midazolam infusions after intubation. She had a transient improvement with positive pressure ventilation and inotropic support, but soon, she exhibited worsening of lactic acidosis, progressive cardiogenic shock, and multiorgan failure. This prompted preparation for ECLS. Before the cannulation procedure could be initiated, she developed bradycardia progressing to cardiac arrest in the presence of the PICU and ECLS teams. ECLS was initiated via femoral cannulation, while high-quality CPR was performed for 27 minutes. This procedure is also known as ECPR. Due to the emergent nature, a distal arterial reperfusion cannula (DPC) could not be placed in the cannulated leg.

**Figure 1:**
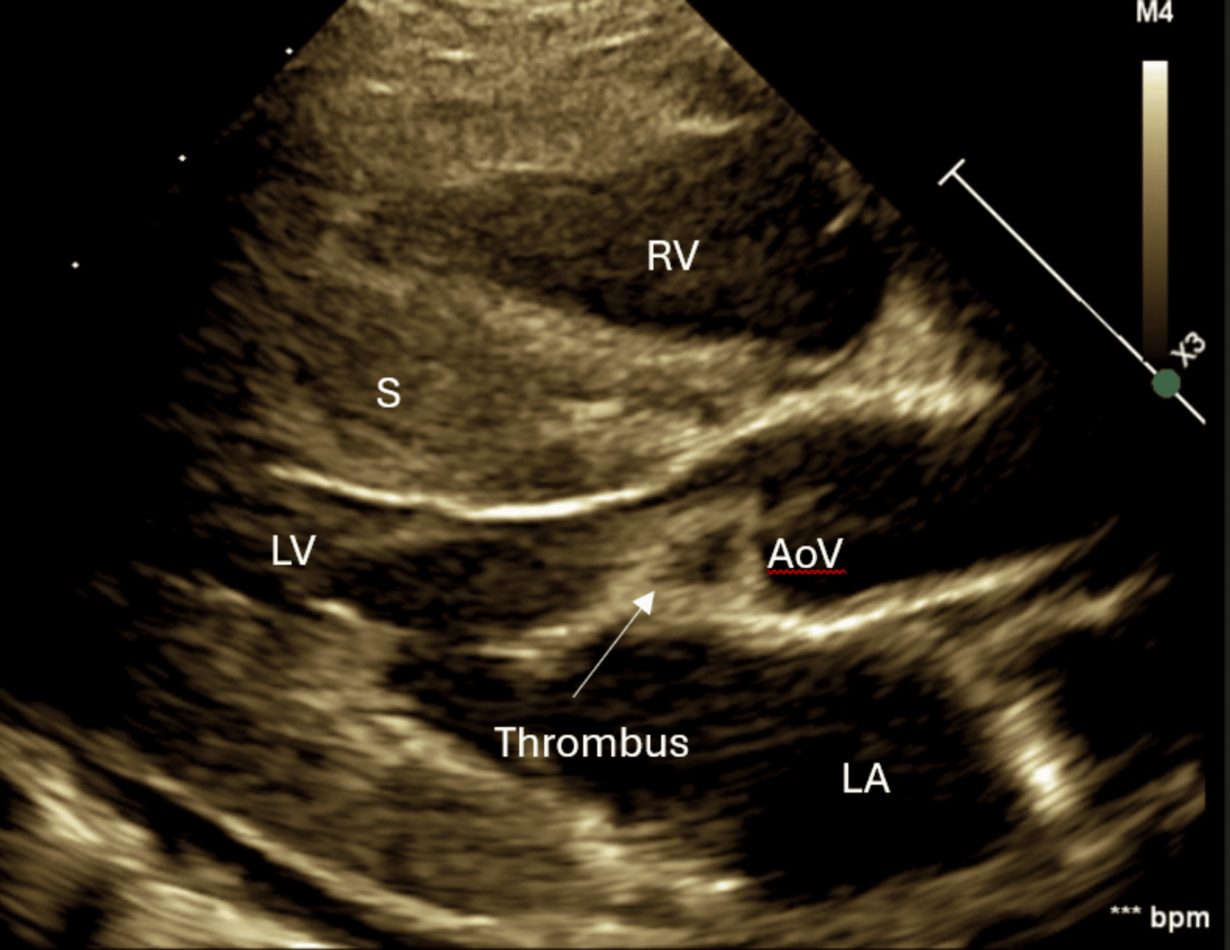
Echocardiogram showing the presence of thrombus RV: right ventricle, S: septum, LV: left ventricle, LA: left atrium, AoV: aortic valve

Post-ECLS, she had no palpable pulses or audible heart sounds. Her echocardiogram showed myocardial stunning, lack of left ventricular (LV) ejection, and opening of the aortic valve. A possible thrombus versus sluggish flow beneath the aortic valve was detected. Troponin was markedly elevated at 33,521 ng/L post-ECPR. Her organ perfusion was maintained with ECLS targeting age-appropriate mean arterial pressure. A repeat echocardiogram 24 hours later (Figure 1) confirmed a 5 × 10 mm thrombus at the LV apex and another moderate-sized thrombus right below the aortic valve measuring 7 mm. Tissue plasminogen activator (TPA) was administered at 0.03 mg/kg/hour for 12 hours. Suspected heparin-induced thrombocytopenia (HIT) prompted an anticoagulation switch from heparin to bivalirudin, and an appropriate workup was sent.

**Figure 2:**
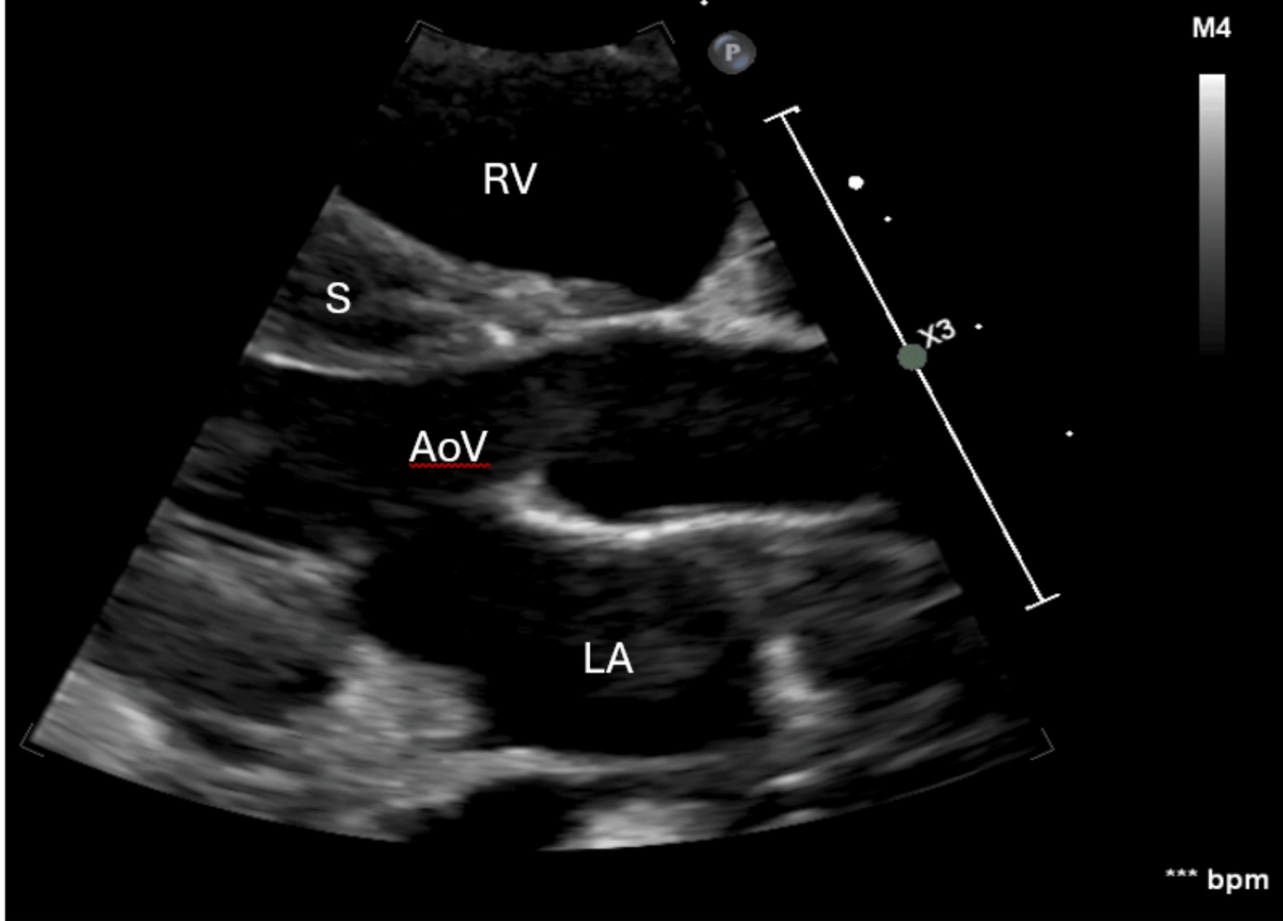
Echocardiogram showing thrombus resolution RV: right ventricle, S: septum, AoV: aortic valve, LA: left atrium

A follow-up echocardiogram 24 hours post-TPA (Figure 2, Video 1) showed complete thrombus resolution, although the left ventricular shortening fraction (LVSF, not LVEF) was only 4%, without ejection and aortic valve opening.

**Video 1:** Echocardiogram showing a presence and then resolution of thrombus with treatment, still with 4% LVSF (not LVEF) RV: right ventricle, S: septum, LV: left ventricle, LA: left atrium, AoV: aortic valve, LVSF: left ventricular shortening fraction, LVEF: left ventricular ejection fraction

She developed right lower extremity (RLE) ischemia, which progressed to compartment syndrome with creatine kinase of 202,830 U/L, requiring fasciotomy. This RLE ischemia was thought to be multifactorial, including a lack of DPC, and then secondary compartment syndrome worsened it further. Continuous renal replacement therapy (CRRT) was initiated for renal support and fluid removal. The other supportive care included parenteral nutrition, antibiotics, and sedation.

Despite these measures, cardiac recovery was minimal, and her lung compliance worsened. She was transferred with ECLS to a quaternary center for transplant evaluation. At the outside institution, the brain computed tomography was unremarkable. Over 20 days, she showed gradual cardiorespiratory recovery and was successfully decannulated from ECLS, but she remained on CRRT. An episode of altered mental status occurred in association with a clotted CRRT circuit. The brain magnetic resonance imaging with angiography (MRI/MRA) revealed an acute-to-early subacute infarction in the right posterior insula and adjacent parietal cortex, along with multiple cerebral and cerebellar microhemorrhages. Her electroencephalogram (EEG) showed diffuse slowing without seizures. The neurological examination normalized quickly. Renal function recovered after seven weeks of CRRT without further need for dialysis or other renal support. She later developed RLE osteomyelitis and foot gangrene, requiring a Syme amputation.

Despite these complications, she was discharged home with a reassuring neurodevelopmental examination. She required heart failure medications (angiotensin-converting enzyme inhibitor and beta-blocker), which were gradually weaned off as her biventricular systolic function completely normalized. She required physical and occupational therapy, tube feeds, and multidisciplinary follow-up.

Follow-up

Approximately eight months later, the patient developed left foot drop and ankle contracture with a transient improvement with rehabilitation. A later emergence of paresthesia suggested the possibility of common peroneal nerve injury. She subsequently developed a left cavovarus foot deformity requiring an orthopedic procedure. Her neurodevelopmental status remained normal. Twenty months after the AFM, her ASD was closed with a percutaneous intervention without further complications.

Currently, she is eight years old and has completed second grade with reassuring neurodevelopmental and cognitive assessments.

## Discussion

Despite a prolonged and complicated course, our patient achieved full cardiac and renal recovery without the need for an external cardiac device or chronic renal replacement therapy. While neuromuscular complications necessitated orthopedic interventions, her neurodevelopmental progress has been excellent. In addition to discussing the meaningful recovery in the context of severe morbidity, we aim to share the key lessons outlined in the conclusion. Here, we share our findings and a relevant literature review.

Clinical presentation and diagnosis of AFM

Acute fulminant myocarditis (AFM) is characterized by myocardial inflammation, often following a viral illness, and can lead to sudden cardiovascular collapse. While histologic criteria exist, clinical diagnosis is widely accepted [10]. Our patient met established criteria, including a viral prodrome, rapidly progressive shock, reduced left ventricular ejection fraction (LVEF) on echocardiography, cardiac arrest, and myocardial stunning for over 48 hours [10-15]. Although her initial LVEF was 66.4% in the setting of cardiac tamponade, it did not improve after pericardiocentesis. Instead, it declined, with no procedural complications observed clinically or on follow-up echocardiography. Taken together, these findings fulfilled the established diagnostic criteria for AFM.

Outcomes and prognostic factors in pediatric AFM and ECPR

Pediatric AFM with shock carries a mortality rate of up to 75% [12], which can be reduced to approximately 20% with timely initiation of extracorporeal life support (ECLS) and meticulous prevention of associated complications [15]. Although the optimal timing for ECLS initiation to prevent the need for extracorporeal cardiopulmonary resuscitation (ECPR) remains undefined, providers should not be dissuaded by its potential necessity, as multiple studies have demonstrated favorable outcomes even with prolonged ECPR and extended ECLS support [4,10-17].

A similar case of pediatric AFM reported the requirement of a biventricular assist device (VAD) after ECPR [16]. In one series of 15 pediatric patients with viral myocarditis, the requirement of ECLS was reported in ten patients, VAD in two patients, and heart transplant in six patients [15]. In another series, six of eight pediatric patients required ECLS, two required VAD, and three patients died from multiple organ failure [17]. While our patient was fortunate not to require VAD or cardiac transplantation, the frequency of such interventions in comparable series highlights the importance of preparedness for advanced supportive measures in pediatric AFM cases.

Increased mortality in pediatric AFM has been associated with female gender, arrhythmias during ECLS, and renal failure requiring dialysis. Variables such as age, weight, race, ECLS era, ventilation duration, or ventilator settings were not predictive of outcomes, including ECPR cases. Favorable outcomes were linked to higher pre-ECLS arterial pH, and the use of sodium bicarbonate or tromethamine was lower in survivors [18]. Our patient’s pre-ECLS arterial pH was 7.26 and a lactate of 6.68, indicating mild to moderate acidosis, yet she experienced a sudden cardiac arrest. Therefore, mild to moderate acidosis in cases of AFM does not safeguard from cardiac arrest.

Neurological complications and recovery

Because of prolonged ECPR, our patient was at higher risk of neurological, cognitive, developmental, and neurovascular complications. She had a normal head CT scan during her course of illness. Post-ECLS MRI showed acute-to-early subacute cerebral infarcts and microhemorrhages. This could be associated with cardiac arrest and prolonged ECPR or as a complication of extended ECLS. It could also be related to CRRT circuit emboli traversing an atrial septal defect, since there was also a clinical correlation at that time with a change in mental status. She also developed right lower extremity (RLE) limb ischemia, complicated by compartment syndrome and osteomyelitis, requiring a Syme amputation. Subsequent left foot cavovarus deformity may reflect peroneal nerve injury or stroke sequelae. The documented incidence of limb ischemia in the setting of femoral V-A ELCS is 52% and can be reduced by selecting appropriate arterial cannula size, establishing a DPC, and monitoring limb perfusion [19]. Even in cases of extracorporeal cardiopulmonary resuscitation (ECPR), a DPC should ideally be inserted as soon as the patient is hemodynamically stabilized. DPC does not resolve this problem completely. Some centers change the cannula site after emergent ECPR, once initial stabilization is complete. Either of these approaches could be considered in such situations, while other strategies need to be sought [19]. Foot drop experienced by our patient has also been reported in the literature [20]. Despite these events, her neurodevelopmental recovery was excellent. Some studies have shown up to 50% intact neurological outcomes after more than an hour of ECPR in isolated cardiac conditions [4]. These results should continue to encourage providers for a potential favorable outcome.

ECLS-related complications and thrombus management

ECLS complications include life-threatening hemorrhage, ischemic stroke, left ventricular distension, thromboembolism, infections, and limb ischemia. Therefore, caution should be practiced to avoid premature ECLS initiation. Our patient developed an intracardiac thrombus, and a low-dose tissue plasminogen activator (TPA) infused over 12 hours achieved a complete intracardiac thrombus resolution. Some reports have shown rapid thrombus clearance with higher doses [21-27] accompanied by a higher risk of fatal hemorrhage [21]. Given the lack of thrombolysis guidelines in the context of ECLS, a low-dose TPA strategy may offer comparable thrombus resolution with lower hemorrhage risk. Bivalirudin, initiated for suspected heparin-induced thrombocytopenia (later excluded), was continued for its efficacy as a primary or secondary anticoagulant in this context [27]. Guidelines regarding anticoagulation on ECMO with bivalirudin are also lacking.

Institutional strategy and preparedness

**Figure 3:**
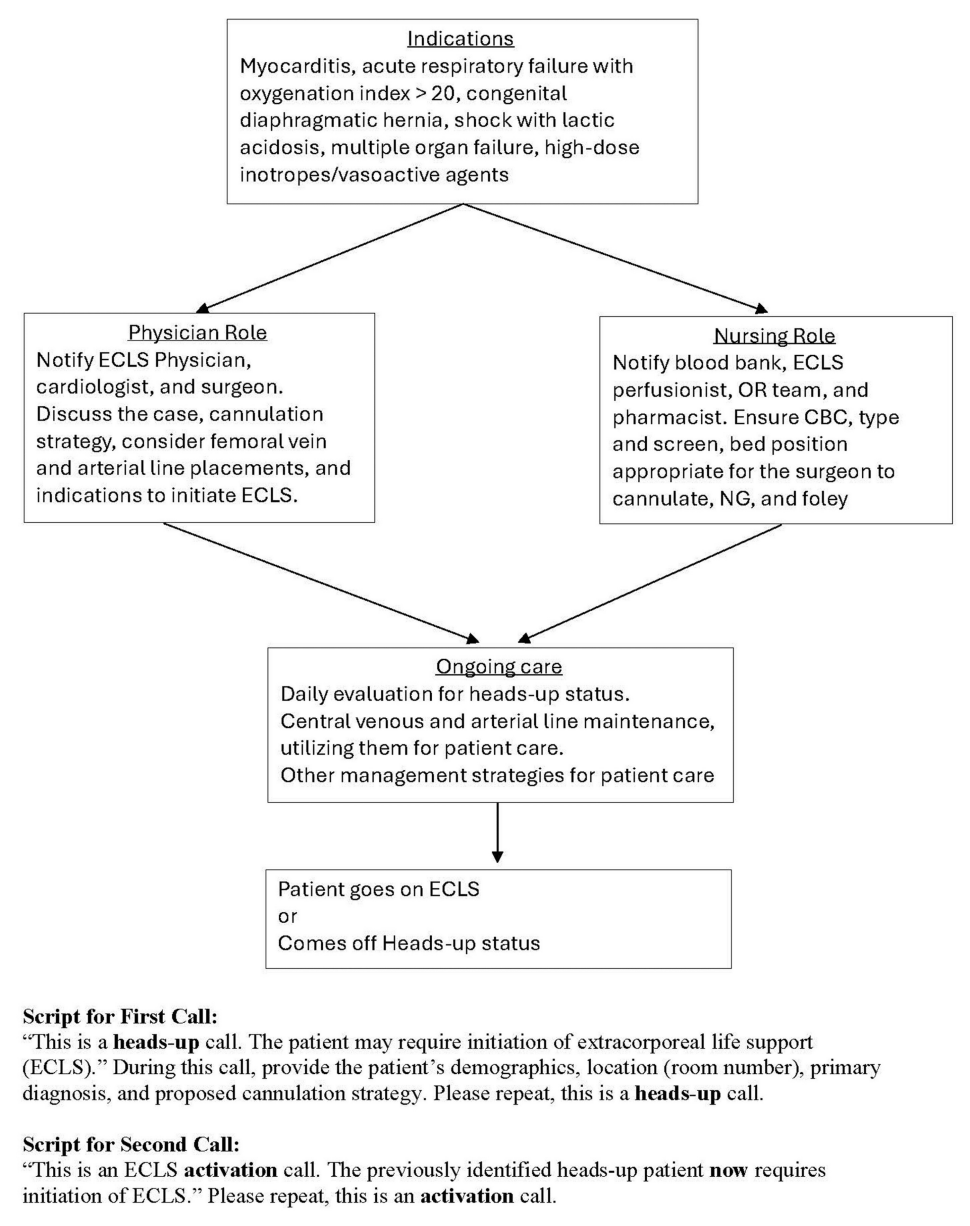
Institutional guidelines for the ECLS heads-up system ECLS: extracorporeal life support, CBC: complete blood count, NG: nasogastric, OR: operating room

Figure 3 illustrates our institutional extracorporeal life support (ECLS) “heads-up” system, which is implemented for high-risk conditions such as myocarditis, severe respiratory failure (including meconium aspiration syndrome, persistent pulmonary hypertension of the newborn, or ARDS with an oxygenation index > 20), congenital diaphragmatic hernia, shock with worsening lactic acidosis, multiple organ failure, or the need for high-dose vasoactive support. Once implemented, physician and nursing leadership assume predefined roles as shown in Figure 3.

One key component of this system is the early placement of femoral central venous and arterial lines in selected pediatric patients. These catheters support routine care and can be rewired for ECLS cannulation, enabling rapid deployment, especially during ongoing CPR, when de novo cannulation is challenging. In this case, although we are not an ECPR-designated center, preemptively placed lines allowed rapid cannulation during cardiac arrest. Another component is ECLS circuit availability at the bedside, using either a dry prime or a saline prime.

These institutional guidelines have been effective and are continually refined. We share them here to provide insight and potential guidance for other centers.

Family perspective

Our daughter was two years old when she embarked on this life-changing journey, which we initially thought was just another “cold.” She was playful and running around, and in the blink of an eye, she was in the ED and then the PICU. She endured a cascade of events, each life-threatening on its own. It began with cardiac tamponade requiring emergent fluid drainage, ECPR for 27 minutes, and a myocardial stun rendering her entirely ECLS-dependent. She developed an intracardiac thrombus and required thrombolytic therapy while already on anticoagulation, placing her at a high risk for catastrophic hemorrhage.

Cannulated in the groin, she developed compartment syndrome of the leg, necessitating emergent fasciotomy. Later, she was painstakingly transferred on ECLS to a tertiary center for heart transplant consideration via transoceanic transport. She remained on ECLS for a total of 20 days and on CRRT for seven weeks. A second intracardiac thrombus and ischemic stroke further complicated her course.

After weeks of fighting for every inch of her leg and foot, it was determined that she would have a Syme amputation of the right foot, allowing her to walk with or without a prosthesis eventually. Months after discharge, it was discovered that her left leg had suffered nerve damage, most likely during her hospitalization, causing a foot drop that would require multiple orthopedic surgeries and recoveries, including relearning how to walk.

Remarkably, she recovered from these overwhelming adversities through extensive rehabilitation over several years. She is now eight years old, attending third grade, with intact cognitive function. She did not require either heart or kidney transplantation. We remain deeply proud of the resilience exhibited by our daughter and our family through this journey, although it has been profoundly emotional to witness her suffering.

Throughout this experience, sustaining hope proved to be our greatest struggle as she adapted to her new realities. Whether relearning how to eat, drink, or walk, each new complication felt insurmountable, any one of which could devastate most families. Although we trusted that the medical teams were relentless and followed best practices, we had to be our daughter’s advocate through every decision, which could have potentially affected the quality of her life or even her survival. That burden weighed heavily every day until only recently, but was buoyed by the caring support of friends, family, and those medical teams.

We hope that sharing our experience encourages both families and healthcare providers to recognize not only the medical complexities but also the emotional journey that accompanies them. Even though each child’s outcome may differ, we hope our story offers strength and reassurance that recovery and resilience remain possible in the most critical circumstances.

## Conclusions

While further research is warranted in the areas discussed in this manuscript, our case report highlights several important considerations. Sudden cardiac arrest is a recognized complication of AFM, emphasizing the need for prompt readiness and support from the medical team. Even after prolonged ECPR and ECLS, complete cardiac recovery and favorable neurodevelopmental outcomes are possible with optimal care. Low-dose TPA may be a feasible option for managing intracardiac thrombus in pediatric patients on ECLS. Centers without ECPR capability can still benefit from structured preparedness systems and early consideration of vascular access. The risk of limb ischemia may be minimized by selecting an appropriate arterial cannula size, implementing a distal perfusion catheter, and monitoring limb perfusion throughout treatment.
